# Cul2 Is Essential for the *Drosophila* IMD Signaling-Mediated Antimicrobial Immune Defense

**DOI:** 10.3390/ijms26062627

**Published:** 2025-03-14

**Authors:** Renjie Duan, Baoyi Hu, Erwen Ding, Shikun Zhang, Mingfei Wu, Yiheng Jin, Umar Ali, Muhammad Abdul Rehman Saeed, Badar Raza, Muhammad Usama, Syeda Samia Batool, Qingshuang Cai, Shanming Ji

**Affiliations:** 1 Center for Developmental Biology, School of Life Sciences, Anhui Agricultural University, Hefei 230036, China; baoyihoo@163.com (B.H.); erwending04522@163.com (E.D.); zsk@stu.ahau.edu.cn (S.Z.); wmf18755985015@163.com (M.W.); jinyh@stu.ahau.edu.cn (Y.J.); umar97ali@stu.ahau.edu.cn (U.A.); mars@stu.ahau.edu.cn (M.A.R.S.); badarraza02@gmail.com (B.R.); musama7339@gmail.com (M.U.); batoolsamia110@outlook.com (S.S.B.); 2 Institut de Génétique et de Biologie Moléculaire et Cellulaire, 67400 Illkirch, France; caiq@igbmc.fr

**Keywords:** Cul2, Eff, IMD signaling pathway, antimicrobial immune defense, *Drosophila melanogaster*

## Abstract

Cullin 2 (Cul2), a core component of the Cullin-RING E3 ubiquitin ligase complex, is integral to regulating distinct biological processes. However, its role in innate immune defenses remains poorly understood. In this study, we investigated the functional significance of Cul2 in the immune deficiency (IMD) signaling-mediated antimicrobial immune reactions in *Drosophila melanogaster* (fruit fly). We demonstrated that loss-of-function of *Cul2* led to a marked reduction in antimicrobial peptide induction following bacterial infection, which was associated with increased fly mortality and bacterial load. The proteomic analysis further revealed that loss-of-function of *Cul2* reduced the expression of Effete (Eff), a key E2 ubiquitin-conjugating enzyme during IMD signaling. Intriguingly, ectopic expression of *eff* effectively rescued the immune defects caused by loss of *Cul2*. Taken together, the results of our study underscore the critical role of *Cul2* in ensuring robust IMD signaling activation, highlighting its importance in the innate immune defense against microbial infection in *Drosophila*.

## 1. Introduction

The innate immune system represents the primary defense mechanism against microbial invasions in all metazoans, functioning through the conserved signaling pathways that detect and neutralize pathogens [1,2,3]. In recent decades, *Drosophila melanogaster* (fruit fly) has been utilized as a pivotal animal model for unraveling the complex regulatory pathways of the host innate immune responses [4,5,6]. Through the conserved signaling cascades and genetically tractable framework, fruit flies provide profound insights into host–pathogen interactions, thereby propelling the frontiers of immunological research. In *Drosophila*, the immune deficiency (IMD) pathway is one of the central components of the innate immune responses, playing a pivotal role in the host defense against some types of Gram-negative bacteria [5,7,8]. Activation of the IMD signaling pathway begins with the recognition of diaminopimelic acid-type peptidoglycans found on the bacterial cell wall. This recognition triggers a series of intracellular signaling events that ultimately activate the nuclear factor kappa B (NF-κB)-like transcription factor Relish (Rel), which drives the expression of several types of antimicrobial peptides (AMPs). These AMPs act as potent effector molecules that directly inhibit bacterial proliferation and survival, ensuring the *Drosophila* immune defenses [8,9,10].

While the core molecular framework of the IMD pathway has been relatively well established, the regulation of this signaling cascade involves numerous accessory factors that are essential for maintaining signal fidelity and efficiency. Among these, ubiquitin-mediated post-translational modifications have emerged as critical mechanisms for fine-tuning IMD signaling [5,8,10,11]. E3 ubiquitin ligases, in particular, play a central role by conferring substrate specificity during ubiquitination, a process that dominantly governs the degradation, activation, or functional modulation of the substrate [12,13]. A series of pioneering investigations have demonstrated that some E3 ligases are integral to specific stages of the IMD signaling pathway, influencing both activation and resolution of the fly antimicrobial immune responses. For instance, during IMD signaling, the key adaptor protein Imd undergoes the 63rd lysine (K63)-linked ubiquitination modification, which is catalyzed mainly by the E3 ligase *Drosophila* inhibitor of apoptosis 2 (Diap2) [14]. Diap2 is also responsible for the K63-linked ubiquitination of some other downstream effectors, including the initiator caspase death-related ced-3/Nedd2-like protein (Dredd) [15] and the inhibitor of κB (IκB) kinase γ (IKKγ), also known as Kenny (Key) [16]. To fulfill the E3 ligase enzymatic activity of Diap2, it needs help from the functional E2 complex consisting of ubiquitin-conjugating enzyme 5 (Ubc5), also known as Effete (Eff), ubiquitin-conjugating enzyme variant 1a (Uev1a), and ubiquitin-conjugating enzyme 13 (Ubc13), also known as Bendless (Ben) [17]. These modifications serve as a docking platform for signaling components, including transforming growth factor-β-activated kinase 1 (Tak1) and the IκB kinase (IKK) complex, which ultimately activate the transcription factor Rel [15,16].

The Cullin-RING E3 ubiquitin ligases (CRLs) constitute a major class of E3 ligases that mediate diverse cellular processes, including cell cycle progression, signal transduction, and protein turnover [18,19,20]. These multi-subunit complexes are built around Cullin family proteins, which serve as scaffolding components for assembling the CRL complex [18,20,21]. We and our collaborators recently performed a genetic screening of the *Drosophila* Cullin family genes in the context of IMD signaling regulation. We identified *Cullin 3* (*Cul3*) as an essential effector for the efficient activation of IMD signaling upon bacterial challenge [22]. In addition, we observed that *Cullin 2* (*Cul2*) would be another potential modulator of the IMD pathway [22]. Although many details of the broad functional scope of *Cul2* in *Drosophila* development have been established, its involvement in the fly antimicrobial immune defense remains poorly understood.

In this study, we addressed the gap in knowledge regarding the functional role of *Cul2* in *Drosophila* innate immunity. By performing a series of genetic approaches, we assessed the impact of *Cul2* on AMP production, bacterial clearance, and fly survival following bacterial infections. The proteomic analysis revealed that loss-of-function of *Cul2* led to a reduction in Eff expression, implicating the involvement of *Cul2* in the regulation of this critical E2 enzyme during IMD signaling. Remarkably, overexpression of *eff* (*eff OE*) was sufficient to rescue the immune defects caused by mutation of *Cul2*, providing evidence of a functional relationship between *Cul2* and *eff* in supporting robust IMD immune signaling. Collectively, our findings highlight a previously unrecognized role of *Cul2* in modulating the IMD pathway and underscore its importance in the fly innate immune defenses against microbial threats. By unraveling the interplay between *Cul2* and *eff*, our study expands the understanding of the molecular mechanisms underlying *Drosophila* immune signaling and provides insights into the broader regulatory landscape of ubiquitin-dependent immune responses.

## 2. Results

### 2.1. Loss-of-Function of Cul2 Prevents AMP Induction in Adult Flies upon Bacterial Stimuli

**Figure 1:**
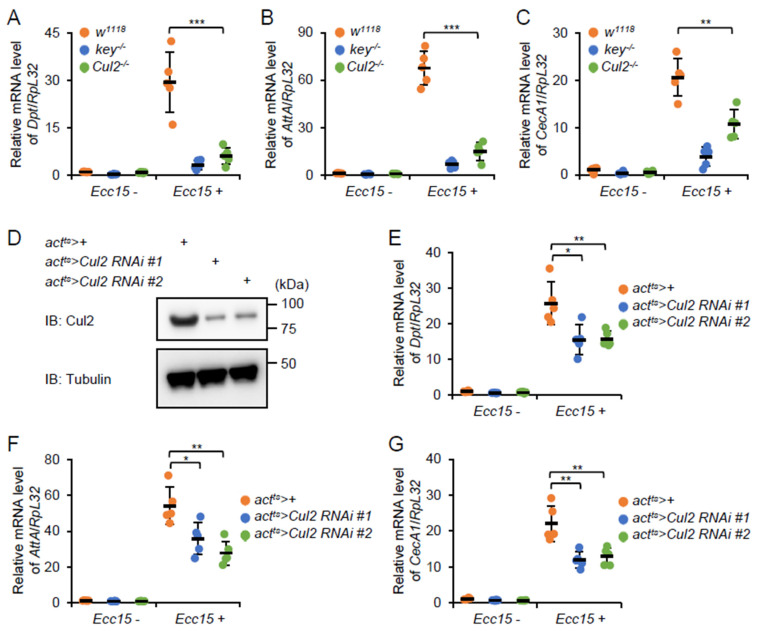
*Cul2* is required for IMD signaling in adult flies after bacterial infections. (**A**–**C**) Age-paired adult flies, including *w^1118^*, *key^-/-^*, and *Cul2^-/-^*, were infected with *Ecc15* (referred to as *Ecc15*+). Twelve hours after infection, flies were homogenized for RT-qPCR assays to monitor the expression levels of *Dpt* (**A**), *AttA* (**B**), and *CecA1* (**C**). Flies without *Ecc15* treatment were referred to as *Ecc15-*. (**D**) Flies, including *act^ts^*>+, *act^ts^*>*Cul2 RNAi #1*, and *act^ts^*>*Cul2 RNAi #2*, were lysed for Western blot experiments. Tubulin was used as loading control. (**E**–**G**) Bacterial infection and RT-qPCR assays were performed as in (**A**–**C**), except that flies used here included *act^ts^*>+, *act^ts^*>*Cul2 RNAi #1*, and *act^ts^*>*Cul2 RNAi #2*. In (**A**–**C**,**E**–**G**), data are shown as mean values plus standard errors. The ANOVA test was used for statistical analyses. *, *p* < 0.05; **, *p* < 0.01; ***, *p* < 0.001.

To investigate the potential involvement of *Cul2* in the *Drosophila* IMD signaling-mediated immune response, we infected the *Cul2^EY09124^* loss-of-function (LOF) mutants (referred to as *Cul2^-/-^*, isogenized with *w^1118^*) and the age-paired *w^1118^* flies (control) with *Pectobacterium carotovorum carotovorum 15* (*Ecc15*). In addition, the isogenized *key^c02831^* LOF mutants (referred to as *key^-/-^*) were also used for *Ecc15* infection in these experimental approaches. *Ecc15* has been widely used to induce IMD signaling in adult flies, which can be easily monitored by looking at the expression levels of the IMD downstream AMP genes, for instance, *Diptericin* (*Dpt*), *Attacin A* (*AttA*), and *Cecropin A1* (*CecA1*) at 12 h post *Ecc15* stimuli [23]. A marked induction of *Dpt*, *AttA*, and *CecA1* was indeed observed in the *w^1118^* control flies 12 h after *Ecc15* infection (Figure 1A–C), suggesting that IMD signaling is activated by *Ecc15* in adult flies. Moreover, the *Ecc15*-induced expressions of these AMPs were drastically prevented in the *key* LOF mutant flies (Figure 1A–C), which were consistent with previous findings [24]. In addition, the transcript levels of *Dpt*, *AttA*, and *CecA1* were decreased by more than 50% in the *Cul2* LOF mutant flies (Figure 1A–C). These data indicate that *Cul2* is required for the robust activation of IMD signaling in adult flies upon bacterial challenge.

To further substantiate the essential role of *Cul2* in mediating the IMD innate immune response, we infected these experimental flies with *Serratia marcescens* (*S. marcescens*), another widely used Gram-negative bacterial pathogen that strongly triggers IMD signaling in adult flies [23]. As demonstrated in Figure S1A–C, the *S. marcescens*-induced upregulations of *Dpt*, *AttA*, and *CecA1* were again decreased in the *Cul2* LOF mutant flies, corroborating the phenotype seen with *Ecc15* infection.

A range of evidence has highlighted the critical role of *Cul2* in modulating *Drosophila* development [25,26,27,28,29]. To confirm that reduced IMD signaling is not an indirect consequence of developmental defects in the *Cul2* LOF mutants, we adopted an adult-specific knockdown strategy using the *act-Gal4;tub-Gal80^ts^* (referred to as *act^ts^*) system. The genetic crosses were first maintained at 18 °C to minimize RNA interference (RNAi) during development and then shifted to 29 °C for 1 w post eclosion to silence *Cul2* expression at the adult stage. We generated two independent RNAi lines, namely, *act^ts^*>*Cul2 RNAi #1* and *act^ts^*>*Cul2 RNAi #2*, and confirmed the knockdown efficiency by Western blot analyses (Figure 1D). Subsequently, we infected the *act^ts^*>*Cul2 RNAi #1*, *act^ts^*>*Cul2 RNAi #2*, and *act^ts^*>+(control) flies with *Ecc15* and examined AMP expressions following the same procedure described above. The inductions of *Dpt*, *AttA*, and *CecA1* were compromised in adult flies with silencing of *Cul2* (Figure 1E–G). Moreover, consistent results were obtained when we used *S. marcescens* for infections, where the IMD-mediated AMP responses were likewise diminished (Figure S1D–F). Collectively, these data demonstrate that *Cul2* is indispensable for the full activation of IMD signaling upon bacterial challenge, independent of the developmental abnormalities that may arise from permanent loss of *Cul2* function.

### 2.2. Cul2 Mediates the Fly Defense Against Bacterial Infection

**Figure 2:**
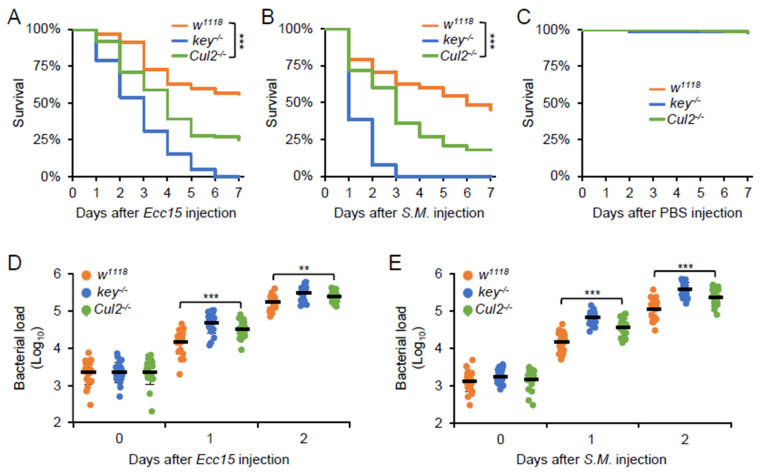
*Cul2* is essential for the fly defense against bacterial infection. (**A**) Adult flies, including *w^1118^*, *key^-/-^*, and *Cul2^-/-^*, were injected with *Ecc15* (**A**), *Serratia marcescens* (*S. M.*, (**B**)), or sterile PBS buffer (**C**). Survival curves of the indicated flies were analyzed. The numbers of flies are as follows. In (**A**), *w^1118^*: 150; *key^-/-^*: 145; *Cul2^-/-^*: 148. In (**B**), *w^1118^*: 149; *key^-/-^*: 146; *Cul2^-/-^*: 145. In (**C**), *w^1118^*: 150; *key^-/-^*: 147; *Cul2^-/-^*: 144. (**D**,**E**) Adult flies, including *w^1118^*, *key^-/-^*, and *Cul2^-/-^*, were injected with *Ecc15* (**D**) or *S. M.* (**E**), followed by bacterial burden assays. In (**A**–**C**), the Log-Rank test was used for statistical analyses. In (**D**,**E**), the ANOVA test was used for statistical analyses. **, *p* < 0.01; ***, *p* < 0.001.

To explore the essential role of *Cul2* in the fly defense against bacterial pathogens, we infected the *Cul2* LOF mutants, the *key* LOF mutants, and the *w^1118^* control flies with *Ecc15* or *S. marcescens.* The *Cul2* LOF mutants exhibited a higher mortality rate compared to the *w^1118^* control flies upon infection with either bacterial species (Figure 2A,B). Notably, no significant difference in survival was observed between the two groups of flies when they were injected with sterile PBS buffer (Figure 2C), indicating that the increased death rate in the *Cul2* LOF flies is directly attributable to the bacterial challenge rather than the injection procedure itself. To further explore whether the heightened susceptibility of the *Cul2* LOF mutants is associated with impaired bacterial clearance, we measured the bacterial burden in these infected flies. As illustrated in Figure 2D,E, the colony-forming unit (CFU) counts of both *Ecc15* and *S. marcescens* were higher in the *Cul2* LOF mutants than those in the *w^1118^* controls, suggesting that loss of *Cul2* disrupts the IMD signaling-mediated induction of key AMPs, thereby resulting in insufficient pathogen clearance and heightened mortality upon infection. Taken together, our data emphasize the crucial role of *Cul2* in orchestrating the effective host defense against bacterial infections in *Drosophila*.

### 2.3. Overexpression of Cul2 Rescues the Immune Defects in Cul2 LOF Mutants

**Figure 3:**
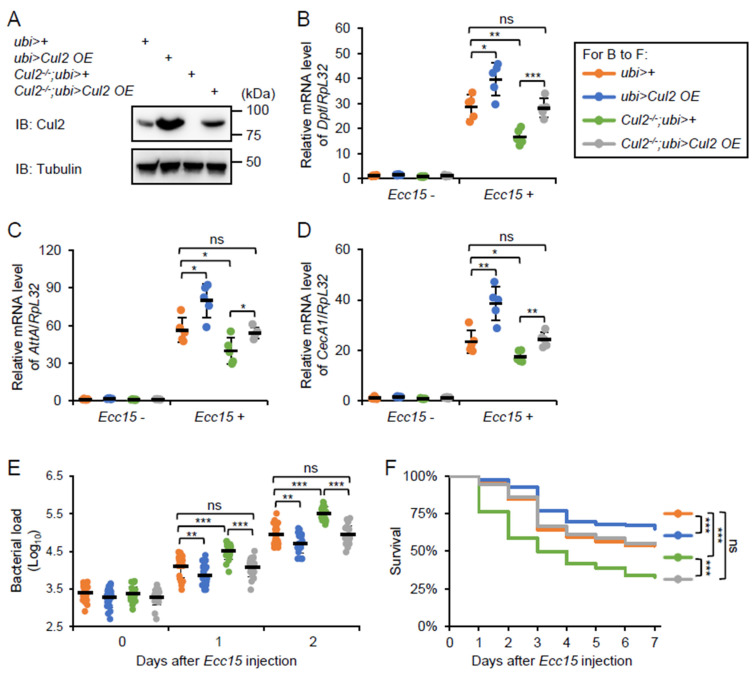
Overexpression of *Cul2* rescues the immune defects in *Cul2* LOF mutants. (**A**) Western blots showing the expression levels of Cul2 in the indicated flies. Tubulin was used as loading control. (**B**–**F**) Adult flies, including *ubi*>+, *ubi*>*Cul2 OE*, *Cul2*^-/-^*; ubi*>+, and *Cul2^-/-^; ubi*>*Cul2 OE* were infected with *Ecc15*, followed by RT-qPCR (**B**–**D**), bacterial burden (**E**), or survival (**F**) assays. In (**F**), the numbers of flies are as follows. *ubi*>+: 148; *ubi*>*Cul2 OE*: 150; *Cul2^-/-^; ubi*>+: 147; *Cul2^-/-^*; *ubi*>*Cul2 OE*: 147. In (**B**–**E**), the ANOVA test was used for statistical analyses. In (**F**), the Log-Rank test was used for statistical analyses. *, *p* < 0.05; **, *p* < 0.01; ***, *p* < 0.001; ns, not significant.

To further confirm the regulatory role of *Cul2* in the *Drosophila* IMD antimicrobial immune defense, we performed rescue experiments by generating a *ubi-Gal4*-driven *Cul2 overexpression* (*Cul2 OE*) strain under the *Cul2^-/-^* genetic background (referred to as *Cul2^-/-^; ubi*>*Cul2 OE*). Similar to the *act-Gal4*, the *ubi-Gal4* is another widely used strain that drives ubiquitous gene expression in *Drosophila*. Indeed, restoration of *Cul2* expression (Figure 3A) rescued the decreases in AMP inductions in the *Cul2* LOF flies after *Ecc15* infection (Figure 3B–D). Moreover, the elevated *Ecc15* burdens in the *Cul2* LOF mutants were reversed by *Cul2 OE* (Figure 3E). Intriguingly, the overall survival of the *Cul2^-/-^;ubi*>*Cul2 OE* flies was comparable to that of the *ubi*>+ control flies (Figure 3F and Figure S2A). These data strongly indicate that *Cul2* is a bona fide modulator in the *Drosophila* immune defense against bacterial infection. Consistently, overexpression of *Cul2* alone benefited the fly immune defense against *Ecc15* infection (Figure 3B–F).

### 2.4. Loss-of-Function of Cul2 Prevents Eff Expression in Adult Flies

**Figure 4:**
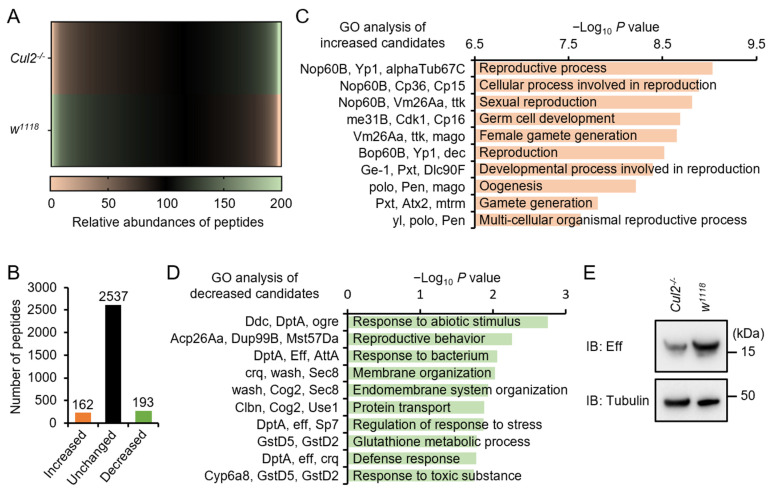
Cul2 mediates the expression of Eff in adult flies. (**A**,**B**) Age-paired *w^1118^* and *Cul2^-/-^* flies were collected for a proteomic analysis (**A**). In (**B**), the number of increased and decreased candidates is shown. (**C**,**D**) GO analyses of increased (**C**) and decreased (**D**) candidates in the proteomic analysis. (**E**) Flies, including *w^1118^* and *Cul2^-/-^*, were subjected to Western blot assays to monitor the expression levels of Eff. Tubulin was used as loading control.

We further explored the molecular mechanism by which *Cul2* contributes to *Drosophila* antimicrobial innate immunity. Since Cullin family proteins are well known for their essential roles in mediating the ubiquitination and turnover of downstream target proteins, we subjected the *Cul2* LOF mutants and *w^1118^* flies to a proteomic analysis (Figure 4A). Around 2892 proteins/peptides were identified via the liquid chromatography–mass spectrometry (LC-MS/MS) assay (Figure 4B). Among them, the abundances of 162 candidates were increased in the *Cul2* LOF mutant flies, while 193 were decreased (Figure 4B and Table S2). We then performed gene ontology (GO) analyses and observed that the upregulated genes were mainly involved in reproduction (Figure 4C), which is consistent with previously reported conclusions regarding *Drosophila Cul2*. On the other hand, the downregulated genes in the *Cul2* LOF mutants fell into categories including “response to abiotic stimulus”, “reproductive behavior”, and “response to bacterium” (Figure 4D). At this stage, we certainly paid close attention to these downregulated candidates and compared their relative abundances in detail. We noted that the levels of several IMD downstream AMPs, for instance, DptA and AttA, were decreased by more than 50% in the *Cul2* LOF mutants (Table S2). Intriguingly, mutation of *Cul2* reduced the protein level of Eff (Table S2), a key E2 ubiquitin-conjugating enzyme in the IMD signaling pathway [14,30]. Meanwhile, we did not observe significant alterations in the context of abundances of other key factors of the IMD signaling pathway (Table S2). We further performed Western blot experiments and confirmed that the protein level of Eff was indeed reduced in the *Cul2* LOF mutants (Figure 4E). Based upon these findings, we propose that Cul2 mediates the fly antimicrobial immune defense through targeting Eff.

### 2.5. Cul2 Modulates Drosophila Antibacterial Immune Defense in an Eff-Dependent Manner

**Figure 5:**
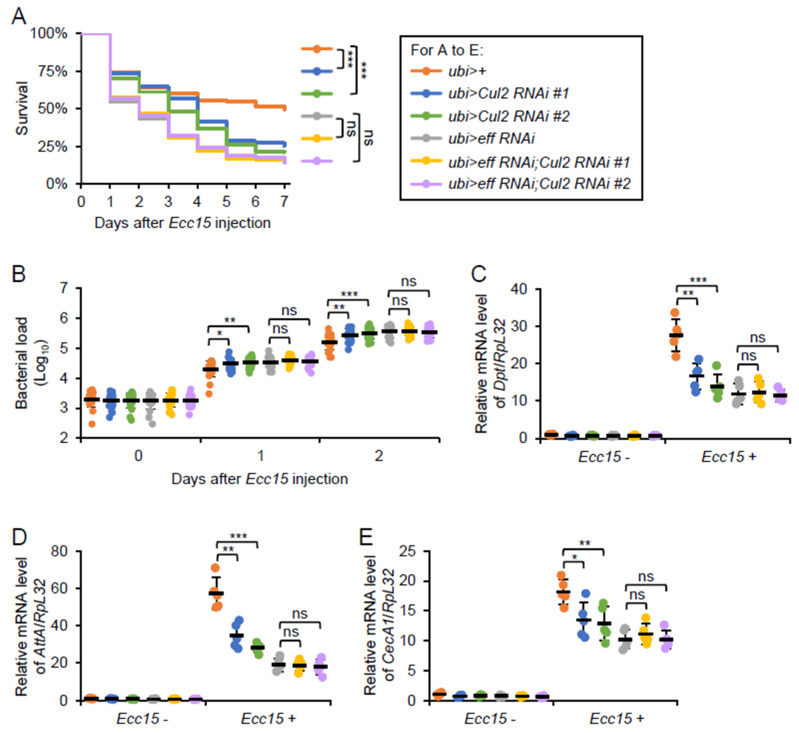
Silencing of *eff* prevents Cul2 effect on immune regulation. (**A**–**E**) Adult flies, including *ubi*>+, *ubi*>*Cul2 RNAi #1*, *ubi*>*Cul2 RNAi #2*, *ubi*>*eff RNAi*, *ubi*>*eff RNAi;Cul2 RNAi #1*, and *ubi*>*eff RNAi;Cul2 RNAi #2*, were infected with *Ecc15*, followed by survival (**A**), bacterial burden (**B**), or RT-qPCR (**C**–**E**) assays. In A, the Log-Rank test was used for statistical analyses. The numbers of flies are as follows. *ubi*>+: 149; *ubi*>*Cul2 RNAi #1*: 147; *ubi*>*Cul2 RNAi #2*: 147; *ubi*>*eff RNAi*: 150; *ubi*>*eff RNAi;Cul2 RNAi #1*: 146; *ubi*>*eff RNAi;Cul2 RNAi #2*: 149. In (**B**–**E**), the ANOVA test was used for statistical analyses. *, *p* < 0.05; **, *p* < 0.01; ***, *p* < 0.001; ns, not significant.

To determine whether *Cul2* modulates the *Drosophila* antibacterial immune defense via the *eff* function, we conducted a series of bacterial infection assays using both genetic knockdown and overexpression approaches. First, we generated flies harboring both *eff RNAi* and *Cul2 RNAi* under the control of *ubi-Gal4*. With PBS treatment, the *eff* and *Cul2* double RNAi flies survived comparably to the controls (*ubi*>+), or the *eff* or *Cul2* single RNAi flies (Figure S2B). However, when we monitored the fly survival rates after *Ecc15* infection, we observed that the *eff RNAi* flies exhibited a decreased survivability, similar to that of the *Cul2 RNAi* flies (Figure 5A). In addition, the *eff* and *Cul2* double RNAi flies did not show a reduction in survival compared to the *eff RNAi* flies alone (Figure 5A), indicating that the requirement of *Cul2* in the fly antibacterial defense is contingent on the presence of functional *eff*. Consistent with this observation, bacterial load measurements revealed that while a single knockdown of *Cul2* led to an enhanced bacterial proliferation in vivo, concomitant knockdown of *eff* essentially masked this *Cul2*-driven effect (Figure 5B), underscoring the *eff*-dependent mode of *Cul2* action. Moreover, silencing of *Cul2* did not reduce the *Ecc15*-induced AMP expression under the *eff RNAi* genetic background (Figure 5C–E). These findings support a model in which *Cul2* modulates the *Drosophila* IMD signaling pathway through *eff*, thereby bolstering the host antibacterial immune response.

**Figure 6:**
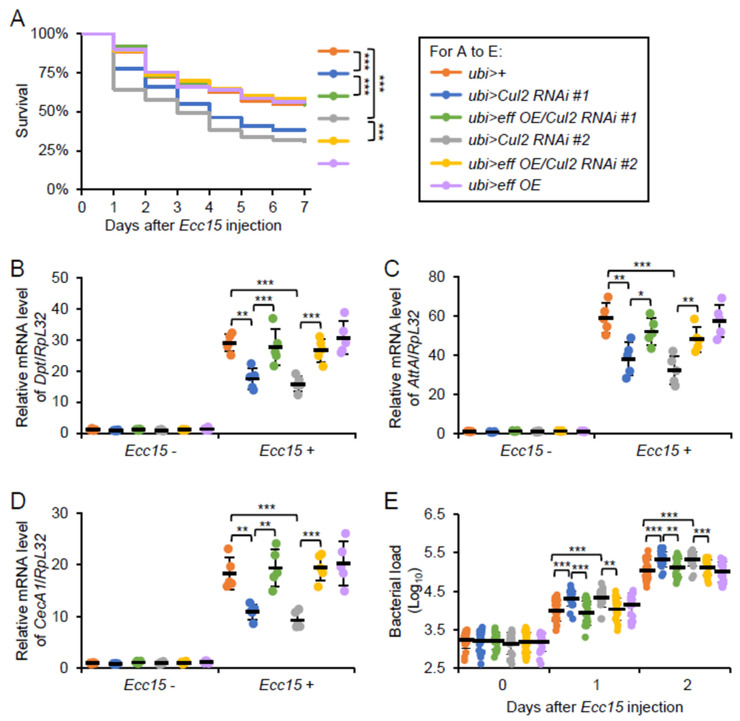
Overexpression of *eff* rescues Cul2 phenotype. (**A**–**E**) Adult flies, including *ubi*>+, *ubi*>*Cul2 RNAi #1*, *ubi*>*eff OE/Cul2 RNAi #1*, *ubi*>*Cul2 RNAi #2*, *ubi*>*eff OE/Cul2 RNAi #2*, and *ubi*>*eff OE*, were infected with *Ecc15*, followed by survival (**A**), bacterial burden (**B**), or RT-qPCR (**C**–**E**) assays. In (**A**), the Log-Rank test was used for statistical analyses. The numbers of flies are as follows. *ubi* > +: 149; *ubi*>*Cul2 RNAi #1*: 147; *ubi*>*eff OE/Cul2 RNAi #1*: 147; *ubi*>*Cul2 RNAi #2*: 150; *ubi*>*eff OE/Cul2 RNAi #2*: 146; *ubi*>*eff OE*: 149. In (**B**–**E**), the ANOVA test was used for statistical analyses. *, *p* < 0.05; **, *p* < 0.01; ***, *p* < 0.001.

We next examined whether heightened *eff* expression could compensate for the immune impairments observed in the *Cul2* LOF mutants. For this, we employed a genetic approach that allowed for *eff overexpression* (*eff OE*) in the context of *Cul2*^-/-^ (referred to as *Cul2^-/-^*; *ubi*>*eff OE*). Following *Ecc15* infection, the *Cul2^-/-^*; *ubi>eff OE* flies displayed a survival rate close to that of the *ubi* > + flies (Figure 6A), even though all of them survived well with PBS treatment (Figure S2C). Furthermore, RT-qPCR assays indicated that *eff OE* restored the downregulation of AMP inductions in the *Cul2* LOF flies (Figure 6B–D). This rescue of AMP expression was consistent with the observation that boosting *eff* level compensated for the absence of functional *Cul2* in modulating bacterial proliferation (Figure 6E). Collectively, our data suggest that *eff* operates downstream of, or at least in close parallel with, *Cul2* in regulating the *Drosophila* antimicrobial immune defense.

## 3. Discussion

Although *Cul2* was initially characterized for its pivotal roles in *Drosophila* development, its potential role in the fly antimicrobial immune defense remains largely unknown. In this study, we demonstrate that *Cul2* is indispensable for mounting an effective antimicrobial defense in adult flies, particularly in response to bacterial challenge. Our findings highlight the broader biological relevance of *Cul2*, underscoring its dual function in both developmental regulation and immune protection in *Drosophila*.

### 3.1. Cul2 Mediates Drosophila Innate Immunity in an Eff-Dependent Manner

**Figure 7:**
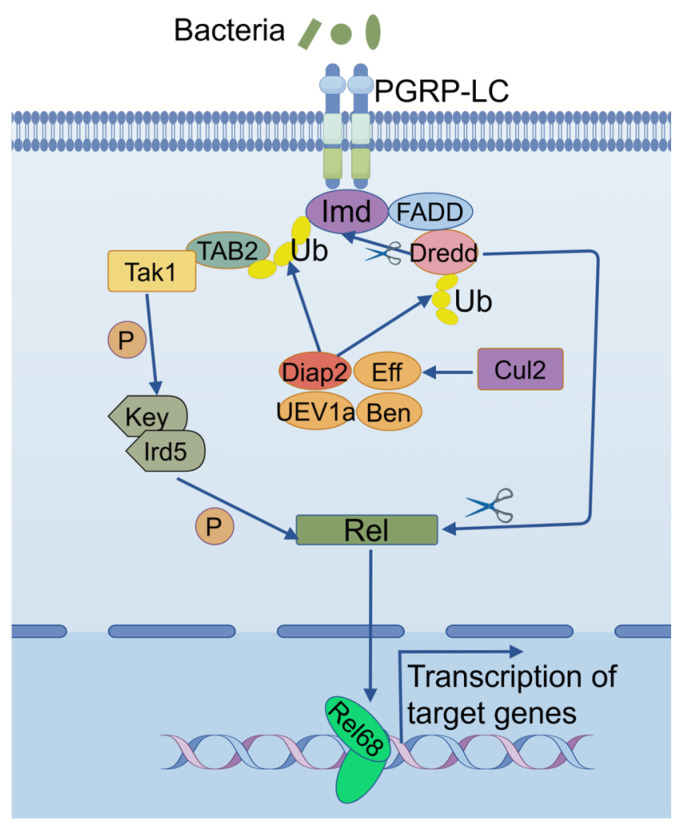
Schematic diagram illustrating the regulatory mechanism involving Cul2-Eff in the Drosophila antibacterial immune defense.

The *Drosophila* Cullin family proteins have been demonstrated to modulate a series of biological processes, especially in the context of development [25,26,27,28,29,31,32,33,34,35,36,37,38]. We previously performed a genetic screening of these proteins and identified that both *Cul2* and *Cul3* play a potential role in regulating IMD signaling [22]. While the immune function of *Cul3* was investigated in detail [22], the immune function of *Cul2* remains unexplored. To address this issue, we first employed the *Cul2* LOF mutant flies for bacterial infections and observed that these mutants exhibit a pronounced susceptibility to bacterial infection, including a reduced survival rate and a diminished expression of key AMPs downstream of the IMD signaling pathway. These phenotypes are reminiscent of defective IMD signaling pathway activity, suggesting that *Cul2* underpins critical signaling events required for an optimal immune response. We next carried out a proteomic analysis and found that mutation of *Cul2* led to a substantial impairment in the expression of Eff, which is one of the key E2-conjugating enzymes responsible for the Diap2-mediated ubiquitination modification of Imd/Dredd during IMD signaling [8,14]. In addition, Eff has also been demonstrated to be involved in modulating development and aging in *Drosophila* [39,40]. Nevertheless, our genetic epistasis analyses further illuminated the relationship between *Cul2* and *eff*. In detail, double RNAi combinations revealed that silencing of *Cul2* in the *eff RNAi* genetic background failed to affect the *Drosophila* immune defenses upon bacterial infection. Conversely, rescue experiments demonstrated that restoring *eff* expression in the *Cul2*-deficient background recovered the fly antimicrobial phenotype, highlighting the downstream function of *eff* in the *Cul2*-dependent regulatory cascade. Collectively, these data underscore the idea that *Cul2* contributes to the *Drosophila* immune surveillance in a manner tightly coupled to *eff* functionality (Figure 7).

### 3.2. Possible Molecular Mechanism by Which Cul2 Regulates Eff Expression

The molecular mechanism by which Cul2 mediates the expression of Eff is a compelling area of investigation, particularly given the well-established role of Cullin family proteins as scaffold factors in the assembly and function of Cullin-RING ubiquitin ligase (CRL) complexes [18,19,21]. These complexes are central to the ubiquitin–proteasome system, which regulates protein stability and turnover, thereby influencing a wide array of cellular processes [19]. In the context of Cul2, it is plausible that this scaffold protein orchestrates the formation of a specific CRL complex by recruiting distinct adaptor proteins, substrate receptors, and E3 ubiquitin ligases. These associated factors collectively determine the specificity of substrate recognition, ubiquitination, and subsequent degradation. However, given that Cul2 positively regulates Eff expression (Figure 4), it is unlikely that Eff itself is a direct substrate for ubiquitination and degradation mediated by Cul2. Instead, a more nuanced mechanism may be at play. We therefore hypothesize that Cul2 modulates the ubiquitination and degradation of a regulatory protein or effector that acts as a repressor of Eff expression. This intermediary effector, when stabilized, would inhibit Eff expression, whereas its degradation, facilitated by Cul2, would relieve this repression, thereby promoting Eff expression. Such a mechanism aligns with the established role of CRL complexes in fine-tuning cellular signaling pathways through the targeted degradation of key regulatory proteins [19,20,41]. To elucidate this proposed mechanism, a combination of co-immunoprecipitation and proteomic analysis would be highly advantageous. Subsequent proteomic profiling could provide a comprehensive map of the ubiquitination targets of the Cul2-containing CRL complex, shedding light on the identity of the putative repressor protein.

On the other hand, an additional layer of regulation might be mediated by circular RNAs (circRNAs), which can serve as molecular scaffolds or sponges for regulatory molecules [42,43,44,45]. Previous studies in mammals have demonstrated that the *Cul2* gene can encode the product of *Cul2 circRNA* to modulate epithelial–mesenchymal transition in hepatocellular carcinoma [46], gastric cancer malignant transformation [47], or colorectal cancer development [48]. These *Cul2* circRNAs exert their regulatory effects primarily by acting as miRNA sponges, sequestering specific miRNAs and thereby preventing them from downregulating their target mRNAs. While the existence and functional roles of *Cul2*-related circRNAs in *Drosophila* remain unexplored, it is plausible that a circRNA derived from the *Cul2* locus could play a similar regulatory role in this model organism. For example, if a *Cul2* circRNA were to sponge a miRNA that normally represses *eff* mRNA translation or stability, this would result in an indirect upregulation of Eff protein levels. This mechanism would add a novel dimension to the understanding of how Cul2 influences Eff expression, extending beyond its canonical role in ubiquitin-mediated proteolysis.

## 4. Materials and Methods

### 4.1. Fly Strain and Husbandry

Flies were reared at 25 °C by using the standard *Drosophila* medium (6.65% cornmeal, 7.15% dextrose, 5% yeast, 0.66% agar, 2.2% nipagin, and 3.4 mL/L propionic acid). The UAS-Gal4 gene expression system was used for the conditional KD/OE of the indicated genes. The *act-Gal4* and *ubi-Gal4* strains can drive ubiquitous gene expression in *Drosophila*. For genetic experiments using the UAS-Gal4 system, crossings were performed at 25 °C. After eclosion, the indicated progenies were transferred to 29 °C and maintained for one week. The *Cul2^EY09124^* (#19883), *eff OE* (#26691), and *ubi*-*Gal4* (#94198) flies were purchased from the Bloomington *Drosophila* Stock Center (Bloomington, IN, USA). The *Cul2 RNAi #2* (#1517) strain was obtained from the Tsinghua Fly Stock Center (Beijing, China). The *eff RNAi* (#105731) strain was purchased from the Vienna *Drosophila* Resource Center (Vienna, Austria). The *Cul2 OE* transgene was generated according to a standard protocol [49]. Briefly, the coding sequence of *Cul2* was first inserted into the *UASp* vector. The *UASp-Cul2* plasmid was further injected into the *w^1118^* embryos together with the *∆^2^*^–*3*^ plasmid. After eclosion, progenies were crossed with the *w^1118^* flies separately for transgene selection. The *Cul2 RNAi #1*, *key^c02831^*, *act-Gal4*, and *tub*-*Gal80^ts^* strains were described previously [50,51,52,53,54].

### 4.2. Bacterial Infection, Survival, and Bacterial Burden Assay

Bacterial infections were carried out as previously described [55,56]. In brief, adult flies were injected with *Ecc15* or *S. marcescens* at a concentration of OD_600_ = 1. The *Ecc15* was a kind gift from Dr. Dominique Ferrandon, and the *S. marcescens* was obtained from the China General Microbiological Culture Collection Center (CGMCC, #1.1215). After bacterial infection, flies were transferred to fresh vials (50 flies per vial). The number of dead flies was scored daily, excluding those that died within 2 h (<5% of the total flies) after injection. Fly survival curves were generated by combining data from 3 independent replicates.

For bacterial burden assays, flies (10 flies for each sample) were harvested and dipped into 75% EtOH. Flies were then volatilized with EtOH on the fly pad for several minutes and homogenized in sterile phosphate-buffered saline (PBS) buffer with serial dilutions. Finally, 100 μL of each dilution was spread on a Luria broth (LB) agar plate at 30 °C overnight. Bacterial clones were scored the next day, and data were collected from 21 independent replicates.

### 4.3. RT-qPCR Assay

Reverse transcription plus quantitative polymerase chain reaction (RT-qPCR) experiments were performed according to a previous protocol [57]. Flies (10 flies for each sample) were collected and homogenized in Trizol reagent (Thermo Fisher, Waltham, MA, USA, Cat#15596018CN) with glass beads. Total RNA was extracted using the standard chloroform/isopropanol method, followed by quality and concentration examination. The cDNA was reverse-transcribed by using the EasyScript First-Strand cDNA Synthesis SuperMix kit (Transgen, Beijing, China, Cat#AE301-02). Quantitative PCR experiments were performed in three technical repetitions by using the TransStart Top Green qPCR SuperMix (Transgen, Cat#AQ131-01). The *RpL32* was used as the internal control. Data were collected from 5 independent biological replicates. Detailed information on primers for RT-qPCR assays is outlined in Table S1.

### 4.4. Western Blot Assay

Western blots were performed as previously described [58,59]. Briefly, flies (50 flies for each sample) were lysed in lysis buffer (150 mM NaCl, 50 mM Tris-HCl, pH = 7.5, 10% glycerol, 0.5% TritonX-100, and 1 mM phenylmethylsulphonyl fluoride). Samples were subjected to centrifugation at high speed for 15 min, and the supernatant was collected for sodium dodecyl sulfate–polyacrylamide gel electrophoresis (SDS-PAGE). After transferring, the PVDF membrane was blocked in PBST (0.1% Tween-20 in PBS) buffer with 5% bovine serum albumin for 30 min. Further, the membrane was incubated with the indicated primary and secondary antibodies. Western blots were revealed by using the enhanced chemiluminescence substrate (Tiangen, Cat#PA112-02). The following antibodies were used for Western blots: mouse anti-β-Tubulin (1:3000, Cowin, Cat#CW0098M); mouse anti-Cul2 (1:1000), which was generated by immunizing mice with the purified fragment of Cul2 (amino acids from 201 to 300); mouse anti-Eff (1:1000), which was generated by immunizing mice with the purified full-length Eff; and goat anti-mouse IgG H&L (1:5000, Abcam, Cambridge, UK, Cat#ab150078).

### 4.5. Proteomic Analysis

The liquid chromatography–mass spectrometry (LC-MS/MS) assay was used for proteomic analysis as we performed previously [60]. In detail, flies (50 flies for each sample) were collected and lysed in lysis buffer (150 mM NaCl, 50 mM Tris-HCl, pH = 7.5, 10% glycerol, 0.5% TritonX-100, and 1 mM phenylmethylsulphonyl fluoride). Samples were precipitated with acetone at 4 °C overnight. The protein pellets were collected and digested with Trypsin (Thermo Fisher, Cat#90057), desalted by using the Pierce C-18 spin column (Thermo Fisher, Cat#89873), and then subjected to LC-MS/MS. The resulting MS/MS data were processed by using the Thermo Proteome Discovery (version 1.4.1.14) software and searched against the UniProt-*Drosophila* database.

### 4.6. Statistical Analysis

Statistical significances were determined by using the ANOVA test in the PASW Statistics 18 software except for fly survival curves, which were analyzed by the Log-Rank test. The number of biological replicates in each assay is appropriate for the indicated statistical analysis. A *p* value of less than 0.05 was considered statistically significant. *, *p*  <  0.05; **, *p*  <  0.01; ***, *p*  <  0.001; ns, not significant.

## 5. Conclusions

In summary, our research reveals the critical role of *Cul2* in *Drosophila* IMD-dependent innate immunity. We propose that *Cul2* achieves this function via targeting Eff, a key E2 ubiquitin-conjugating enzyme in the IMD signaling pathway. Our findings not only broaden our understanding of the immune function of *Cul2* in *Drosophila* but also resonate with the multifaceted roles of *Cul2* in mammals, particularly in orchestrating responses to environmental stresses and pathogenic challenges. By illuminating these conserved mechanisms, our study provides a foundation for future investigations into how *Cul2* and its regulatory circuits can be harnessed or manipulated to bolster immune defense in both invertebrates and vertebrates.

## Data Availability

The data generated in this study are available upon request.

## Supplementary Materials

The following supporting information can be downloaded at https://www.mdpi.com/article/10.3390/ijms26062627/s1.

## References

[1] Beutler B. (2004). Innate immunity: An overview. Mol. Immunol..

[2] Akira S., Uematsu S., Takeuchi O. (2006). Pathogen recognition and innate immunity. Cell.

[3] Janeway C.A., Medzhitov R. (2002). Innate immune recognition. Annu. Rev. Immunol..

[4] Hoffmann J.A. (2003). The immune response of *Drosophila*. Nature.

[5] Yu S., Luo F., Xu Y., Zhang Y., Jin L.H. (2022). *Drosophila* Innate Immunity Involves Multiple Signaling Pathways and Coordinated Communication Between Different Tissues. Front. Immunol..

[6] Ferrandon D., Imler J.L., Hoffmann J.A. (2004). Sensing infection in *Drosophila*: Toll and beyond. Semin. Immunol..

[7] Buchon N., Silverman N., Cherry S. (2014). Immunity in *Drosophila melanogaster*--from microbial recognition to whole-organism physiology. Nat. Rev. Immunol..

[8] Kleino A., Silverman N. (2014). The *Drosophila* IMD pathway in the activation of the humoral immune response. Dev. Comp. Immunol..

[9] Lemaitre B., Hoffmann J. (2007). The host defense of *Drosophila melanogaster*. Annu. Rev. Immunol..

[10] Myllymaki H., Valanne S., Ramet M. (2014). The *Drosophila* imd signaling pathway. J. Immunol..

[11] Goto A., Ji S., Chtarbanova S., Kuraishi T. (2023). Editorial: Inflammatory and inflammatory-like responses in insects. Front. Immunol..

[12] Zheng N., Shabek N. (2017). Ubiquitin Ligases: Structure, Function, and Regulation. Annu. Rev. Biochem..

[13] Swatek K.N., Komander D. (2016). Ubiquitin modifications. Cell Res..

[14] Paquette N., Broemer M., Aggarwal K., Chen L., Husson M., Erturk-Hasdemir D., Reichhart J.M., Meier P., Silverman N. (2010). Caspase-mediated cleavage, IAP binding, and ubiquitination: Linking three mechanisms crucial for *Drosophila* NF-kappaB signaling. Mol. Cell.

[15] Meinander A., Runchel C., Tenev T., Chen L., Kim C.H., Ribeiro P.S., Broemer M., Leulier F., Zvelebil M., Silverman N. (2012). Ubiquitylation of the initiator caspase DREDD is required for innate immune signalling. EMBO J..

[16] Aalto A.L., Mohan A.K., Schwintzer L., Kupka S., Kietz C., Walczak H., Broemer M., Meinander A. (2019). M1-linked ubiquitination by LUBEL is required for inflammatory responses to oral infection in *Drosophila*. Cell Death. Differ..

[17] Chen L., Paquette N., Mamoor S., Rus F., Nandy A., Leszyk J., Shaffer S.A., Silverman N. (2017). Innate immune signaling in *Drosophila* is regulated by transforming growth factor beta (TGFbeta)-activated kinase (Tak1)-triggered ubiquitin editing. J. Biol. Chem..

[18] Petroski M.D., Deshaies R.J. (2005). Function and regulation of cullin-RING ubiquitin ligases. Nat. Rev. Mol. Cell Biol..

[19] Sarikas A., Hartmann T., Pan Z.Q. (2011). The cullin protein family. Genome Biol..

[20] Harper J.W., Schulman B.A. (2021). Cullin-RING Ubiquitin Ligase Regulatory Circuits: A Quarter Century Beyond the F-Box Hypothesis. Annu. Rev. Biochem..

[21] Zimmerman E.S., Schulman B.A., Zheng N. (2010). Structural assembly of cullin-RING ubiquitin ligase complexes. Curr. Opin. Struct. Biol..

[22] Kong F., Wang Z., Zhang C., Xiao Y., Saeed M.A.R., Li W., Goto A., Cai Q., Ji S. (2025). *Drosophila* Cul3 contributes to Diap2-mediated innate immune signaling for antimicrobial defense. hLife.

[23] Neyen C., Bretscher A.J., Binggeli O., Lemaitre B. (2014). Methods to study *Drosophila* immunity. Methods.

[24] Rutschmann S., Jung A.C., Zhou R., Silverman N., Hoffmann J.A., Ferrandon D. (2000). Role of *Drosophila* IKK gamma in a toll-independent antibacterial immune response. Nat. Immunol..

[25] Cai Q., Yan J., Duan R., Zhu Y., Hua Y., Liao Y., Li Q., Li W., Ji S. (2023). E3 ligase Cul2 mediates *Drosophila* early germ cell differentiation through targeting Bam. Dev. Biol..

[26] Ayyub C., Banerjee K.K., Joti P. (2015). Reduction of Cullin-2 in somatic cells disrupts differentiation of germline stem cells in the *Drosophila* ovary. Dev. Biol..

[27] Ayyub C. (2011). Cullin-5 and cullin-2 play a role in the development of neuromuscular junction and the female germ line of *Drosophila*. J. Genet..

[28] Qian Y., Ng C.L., Schulz C. (2015). CSN maintains the germline cellular microenvironment and controls the level of stem cell genes via distinct CRLs in testes of *Drosophila melanogaster*. Dev. Biol..

[29] Monahan A.J., Starz-Gaiano M. (2015). Socs36E limits STAT signaling via Cullin2 and a SOCS-box independent mechanism in the *Drosophila* egg chamber. Mech. Dev..

[30] Park E.S., Elangovan M., Kim Y.J., Yoo Y.J. (2016). UbcD4, an ortholog of E2-25K/Ube2K, is essential for activation of the immune deficiency pathway in *Drosophila*. Biochem. Biophy. Res. Commun..

[31] Hayden L., Chao A., Deneke V.E., Vergassola M., Puliafito A., Di Talia S. (2022). Cullin-5 mutants reveal collective sensing of the nucleocytoplasmic ratio in *Drosophila* embryogenesis. Curr. Biol..

[32] Hudson A.M., Cooley L. (2010). *Drosophila* Kelch functions with Cullin-3 to organize the ring canal actin cytoskeleton. J. Cell Biol..

[33] Tare M., Chimata A.V., Gogia N., Narwal S., Deshpande P., Singh A. (2020). An E3 ubiquitin ligase, cullin-4 regulates retinal differentiation in *Drosophila* eye. Genesis.

[34] Mistry H., Wilson B.A., Roberts I.J., O’Kane C.J., Skeath J.B. (2004). Cullin-3 regulates pattern formation, external sensory organ development and cell survival during *Drosophila* development. Mech. Dev..

[35] Hudson A.M., Mannix K.M., Cooley L. (2015). Actin cytoskeletal organization in *Drosophila* germline Ring Canals Depends on Kelch Function in a Cullin-RING E3 Ligase. Genetics.

[36] Kim S.H., Kim H.J., Kim S., Yim J. (2010). Drosophila Cand1 regulates Cullin3-dependent E3 ligases by affecting the neddylation of Cullin3 and by controlling the stability of Cullin3 and adaptor protein. Dev. Biol..

[37] Baker N.E., Bhattacharya A., Firth L.C. (2009). Regulation of Hh signal transduction as *Drosophila* eye differentiation progresses. Dev. Biol..

[38] Kugler J.M., Lem C., Lasko P. (2010). Reduced cul-5 activity causes aberrant follicular morphogenesis and germ cell loss in *Drosophila* oogenesis. PLoS ONE.

[39] Cipressa F., Cenci G. (2013). Effete, an E2 ubiquitin-conjugating enzyme with multiple roles in *Drosophila* development and chromatin organization. Fly.

[40] Hunt L.C., Curley M., Nyamkondiwa K., Stephan A., Jiao J., Kavdia K., Pagala V.R., Peng J., Demontis F. (2025). The ubiquitin-conjugating enzyme UBE2D maintains a youthful proteome and ensures protein quality control during aging by sustaining proteasome activity. PLoS Biol..

[41] Duda D.M., Scott D.C., Calabrese M.F., Zimmerman E.S., Zheng N., Schulman B.A. (2011). Structural regulation of cullin-RING ubiquitin ligase complexes. Curr. Opin. Struct. Biol..

[42] Kristensen L.S., Andersen M.S., Stagsted L.V.W., Ebbesen K.K., Hansen T.B., Kjems J. (2019). The biogenesis, biology and characterization of circular RNAs. Nat. Rev. Genet..

[43] Li H. (2023). circRNA: A promising all-around star in the future. Epigenomics.

[44] Zhou W.Y., Cai Z.R., Liu J., Wang D.S., Ju H.Q., Xu R.H. (2020). Circular RNA: Metabolism, functions and interactions with proteins. Mol. Cancer.

[45] Zhao X., Zhong Y., Wang X., Shen J., An W. (2022). Advances in Circular RNA and Its Applications. Int. J. Med. Sci..

[46] Meng J., Chen S., Han J.X., Qian B., Wang X.R., Zhong W.L., Qin Y., Zhang H., Gao W.F., Lei Y.Y. (2018). Twist1 Regulates Vimentin through Cul2 Circular RNA to Promote EMT in Hepatocellular Carcinoma. Cancer Res..

[47] Peng L., Sang H., Wei S., Li Y., Jin D., Zhu X., Li X., Dang Y., Zhang G. (2020). circCUL2 regulates gastric cancer malignant transformation and cisplatin resistance by modulating autophagy activation via miR-142-3p/ROCK2. Mol. Cancer.

[48] Yang B.L., Liu G.Q., Li P., Li X.H. (2022). Circular RNA CUL2 regulates the development of colorectal cancer by modulating apoptosis and autophagy via miR-208a-3p/PPP6C. Aging.

[49] Hu Y., Kong F., Guo H., Hua Y., Zhu Y., Zhang C., Qadeer A., Xiao Y., Cai Q., Ji S. (2024). *Drosophila* eIF3f1 mediates host immune defense by targeting dTak1. EMBO Rep..

[50] Ji S., Sun M., Zheng X., Li L., Sun L., Chen D., Sun Q. (2014). Cell-surface localization of Pellino antagonizes Toll-mediated innate immune signalling by controlling MyD88 turnover in *Drosophila*. Nat. Commun..

[51] Zhu Y., Liu L., Zhang C., Zhang C., Han T., Duan R., Jin Y., Guo H., She K., Xiao Y. (2023). Endoplasmic reticulum-associated protein degradation contributes to Toll innate immune defense in *Drosophila melanogaster*. Front. Immunol..

[52] Zhang C., Zhang S., Kong F., Xiao Y., She K., Jin Y., Li J., Qadeer A., Zheng X., Ji S. (2023). Ubiquitin C-terminal hydrolase L5 plays an essential role in the fly innate immune defense against bacterial infection. Front. Biosci..

[53] Ji S., Hoffmann J.A. (2024). Toll-9 prevents the proliferation of injected oncogenic cells in adult flies. J. Genet. Genom..

[54] Cai Q., Wang Z., Xiao Y., Zhang C., Yang Y., Kong F., Feng Y., Guo H., Saeed M.A.R., Ali U. (2025). MESR4 targets bam to mediate intestinal homeostasis and aging in adult flies. Insect Sci..

[55] Cai Q., Guo H., Fang R., Hua Y., Zhu Y., Zheng X., Yan J., Wang J., Hu Y., Zhang C. (2022). A Toll-dependent Bre1/Rad6-cact feedback loop in controlling host innate immune response. Cell Rep..

[56] Hua Y., Zhu Y., Hu Y., Kong F., Duan R., Zhang C., Zhang C., Zhang S., Jin Y., Ye Y. (2022). A feedback regulatory loop involving dTrbd/dTak1 in controlling IMD signaling in *Drosophila melanogaster*. Front. Immunol..

[57] Kong F., Qadeer A., Xie Y., Jin Y., Li Q., Xiao Y., She K., Zheng X., Li J., Ji S. (2023). Dietary supplementation of aspirin promotes *Drosophila* defense against viral infection. Molecules.

[58] Ji S., Zhou X., Hoffmann J.A. (2024). Toll-mediated airway homeostasis is essential for fly survival upon injection of RasV12-GFP oncogenic cells. Cell Rep..

[59] Zhu Y., Cai Q., Zheng X., Liu L., Hua Y., Du B., Zhao G., Yu J., Zhuo Z., Xie Z. (2021). Aspirin positively contributes to *Drosophila* intestinal homeostasis and delays aging through targeting Imd. Aging Dis..

[60] Zheng X., Jin Y., Zhang C., Zhu Y., Guo H., Duan R., Xiao Y., Hu B., Yang Y., Ding E. (2025). RNA-binding protein Roq modulates the *Drosophila* STING antiviral immune response. Cell Investig..

