# Supplementary material for: Cul2 Is Essential for the Drosophila IMD Signaling-Mediated Antimicrobial Immune Defense
Source: Int J Mol Sci. 2025 Mar 14;26(6):2627. doi: 10.3390/ijms26062627 (PMC11941880; doi:10.3390/ijms26062627)
Supplement: Supplementary file 1 [file ijms-26-02627-s001.zip › Supplementary Figure.pdf]

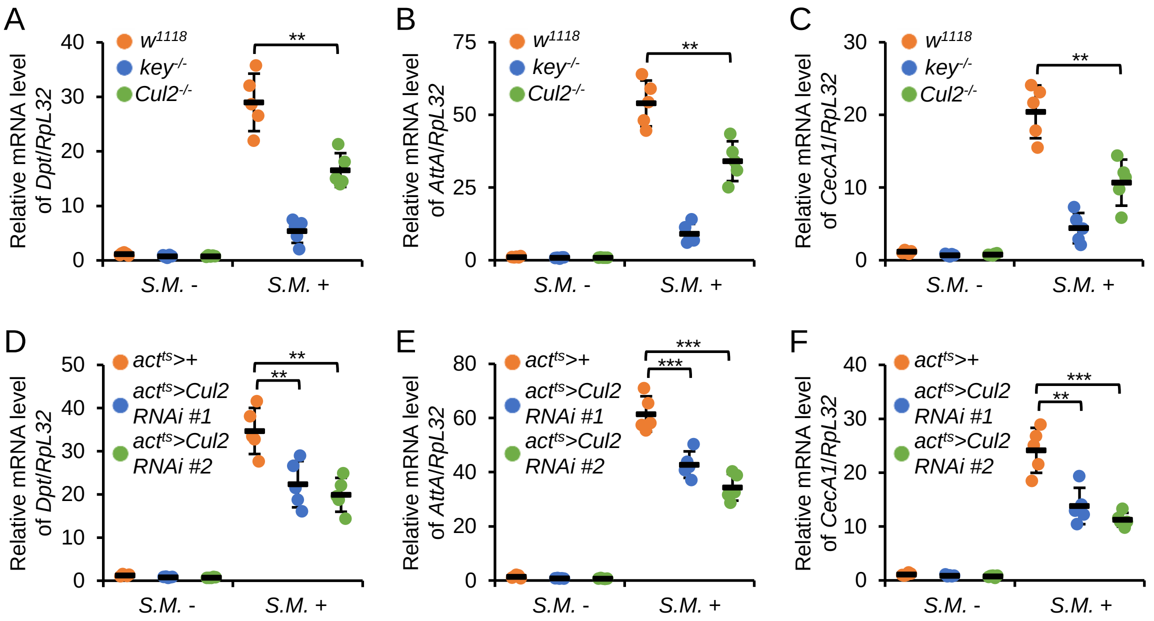

Supplementary Figure S1. Loss-of-function of *Cul2* impairs AMP inductions in adult flies after bacterial infection. (A-C) Adult flies, including w^{1118}, $key^{-/-}$, and $Cul2^{-/-}$ were infected with *S. marcescens* (referred to as *S.M.* +), followed by RT-qPCR assays to monitor the expression profiles of *Dpt* (A), *AttA* (B), and *CecA1* (C). Flies without bacterial infection (referred to as *S.M.* -) were collected as controls. (D-F) Bacterial infections and RT-qPCR assays were performed as in A-C, except that flies used here were act^{ts}>+, act^{ts}>*Cul2 RNAi #1,* and act^{ts}>*Cul2 RNAi #2*. In A-F, the ANOVA test was used for statistical analyses. **, $P < 0.01$; ***, $P < 0.001$.

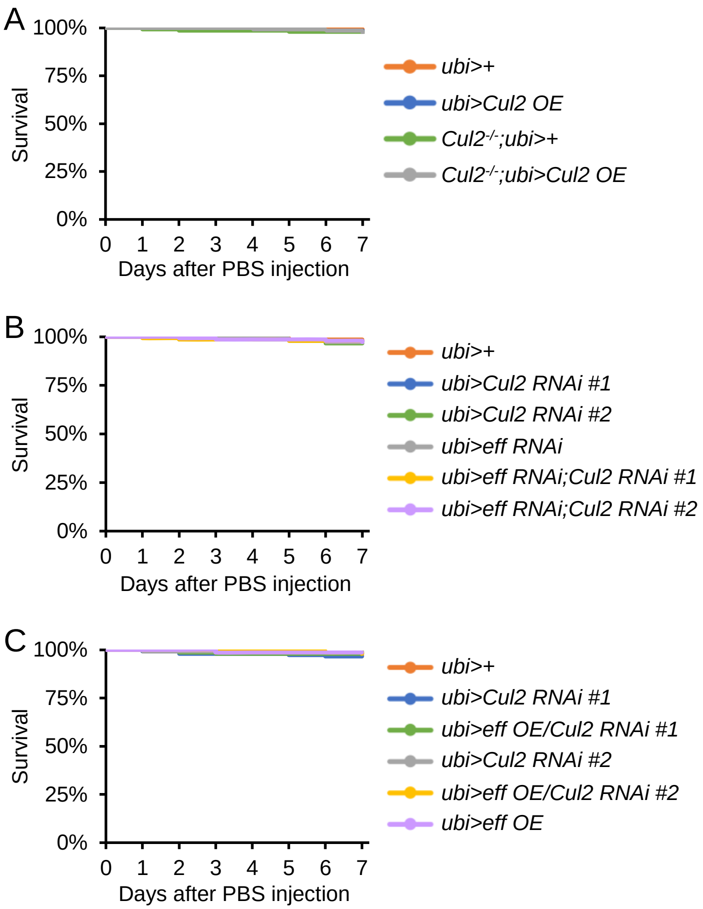

Supplementary Figure S2. Survival curves of various flies with PBS treatment.
(A-C) Indicated adult flies were injected with sterile PBS buffer, followed by survival assays. The numbers of flies are as follows. In A, *ubi*>+: 150; *ubi>Cul2 OE*: 149; *$Cul2^{-/-}$*;*ubi*>+: 150; *$Cul2^{-/-}$;ubi>Cul2 OE*: 146. In B, *ubi*>+: 146; *ubi>Cul2 RNAi #1*: 148; *ubi>Cul2 RNAi #2*: 149; *ubi>eff RNAi*: 148; *ubi>eff RNAi;Cul2 RNAi #1*: 148; ; *ubi>eff RNAi;Cul2 RNAi #2*: 150. In C, *ubi*>+: 150; *ubi>Cul2 RNAi #1*: 148; *ubi>eff OE;Cul2 RNAi #1*: 150; *ubi>Cul2 RNAi #2*: 150; *ubi>eff OE;Cul2 RNAi #2:* 147; *ubi>eff OE*: 149.
